# The Ability of Ultrasound Sonography (USG) to Detect Intrauterine Growth Restriction (IUGR) in the Third Trimester of Pregnancy With the Gold Standard of IUGR (Parameters by USG Hadlock) as a Diagnostic Criterion

**DOI:** 10.7759/cureus.20523

**Published:** 2021-12-19

**Authors:** Zunaira Rafique, Muhammad Wasim Awan, Shaghaf Iqbal, Naila Nasir Usmani, Mashkoor Ahmad, Maryam Amjad, SamiI Ullah, Hassan Mumtaz

**Affiliations:** 1 Diagnostic Radiology, Khan Research Laboratories (KRL) Hospital, Islamabad, PAK; 2 Diagnostic Radiology, KRL Hospital, Islamabad, PAK; 3 Internal Medicine, Bahria International Hospital, Rawalpindi, PAK; 4 Public Health, Health Services Academy, Islamabad, PAK; 5 Forensic Medicine, Riphah International University, Islamabad, PAK; 6 General Medicine, Surrey Docks Health Center, London, GBR; 7 Urology, Guys & St Thomas Hospital, London, GBR

**Keywords:** hadlock, iugr, gestational age, placental thickness, ultrasound

## Abstract

Objective

To investigate the diagnostic accuracy of the placental thickness measured by ultrasound sonography test (USG) in detecting intrauterine growth restriction (IUGR) babies in the third trimester of pregnancy, keeping IUGR (by parameters using Hadlock) as the gold standard.

Methods and materials

This cross-sectional study was conducted at the radiology department of KRL Hospital from August 5, 2020, to October 25, 2021. Informed written consent was also obtained from each patient, and the hospital's ethical committee approved the study. Three hundred and sixty-two (N=362) pregnant women patients knowing of their last menstrual period, age group 20-35 years, BMI usual, and 24 weeks gestation were included. The patient's complete history was taken by clinical examination and then ultrasound was carried out to measure the placental thickness. At 24, 32, and 36 weeks, the thickness of the placenta was assessed. The Hadlock method was used to compute the predicted fetal weight by measuring biparietal diameter (BPD), head circumference (HC), abdominal circumference (AC), and femur length (FL) on the GC Logiq P/6 three-dimensional machine (GE, Tampa, FL). SPSS v 23 (IBM Corp., Armonk, NY) was used to calculate the mean and standard deviation from the collected data.

Results

A total of 362 patients who presented in the radiology department for antenatal ultrasound in the third trimester were recruited in our study. The mean age was 27.26 ± 4.21 years (20-35 years). In our study, the mean placenta thickness at 24 gestation weeks was 24.55 ± 0.79 mm, at 32 gestation weeks was 31.84 ± 1.34 mm, and at 36 gestation weeks was 35.54 ± 2.78. Thus, ultrasound's sensitivity, specificity, positive predictive value, and negative predictive value to determine IUGR by placental thickness was 86.30%, 86.70%, 75%, and 92%, respectively. The diagnostic accuracy of ultrasound incorrectly estimating low placental thickness was 86.40%.

Conclusion

Between 24 and 36 weeks of pregnancy, placental thickness rises almost linearly. As a result, measuring placental thickness and other factors is critical for estimating fetal age, particularly in the late second and early third trimesters, when the exact duration of pregnancy is uncertain. Placentas that were less than 29 mm thick at 32 weeks and 31 mm thick at 36 weeks were related to higher morbidity, lower Apgar scores, and more nursery admissions.

## Introduction

Intrauterine growth restriction (IUGR) is described as a fetus with a birth weight that is significantly lower than the tenth percentile (the lower limit of average weight) for the gestational age [1]. IUGR may be caused by various maternal or fetal factors, with the placenta being one of the most common causes of growth restriction. The prominent role of the placenta is to provide nutrients through a healthy uteroplacental system [2].

Three critical factors contribute to a healthy baby's growth at term: genetics, a healthy mother, and a well-functioning uteroplacental system. The most vital element is the placenta. Unfortunately, it is often ignored [1-3]. The standard weight of the placenta at term in a normal pregnancy is approximately 1/5th of the fetal weight [4]. In utero life, both the placenta and fetus are subjected to the same stress. The fetus and placenta would be affected by any maternal disease. As a result, placental measures, such as thickness, must represent the fetus's condition and outcome [5].

Any placental development problems may significantly affect fetal growth and yield. Since the 1940s, the ratio of fetal birth weight to placental weight has been used to predict normal fetal development [6]. At nine to 10 gestational weeks, an ultrasound revealed the definitive placenta with a uniform granular echogenic outline. Ultrasound (US) allows for placental assessment and identification of various defects using a variety of variables such as thickness and length [7].

Placental thickness abnormality arises due to the uncertainty of the underlying pathological process. Chromosomal defects, preeclampsia, chronic fetal infections, intrauterine growth restriction, and diabetes have been linked to the small placenta [8]. Several studies have found a connection between a small placenta and low birth weight (LBW), as well as IUGR [9]. Some IUGR pregnancies are constitutionally small but healthy; however, others fail to reach their full growth potential due to factors that influence growth like malnutrition, chromosomal abnormalities, medications, or infections. Prenatal detection of IUGR is critical for a better perinatal outcome [10-11].

Placental thickness anomalies with the corresponding gestational age (GA) are one of the early warning signs for developing IUGR "according to the hypothesis that reduced placental size precedes the onset of IUGR." The use of ultrasound (US) Hadlock for placental size assessment is a secure, simple, inexpensive, feasible, and non-invasive diagnostic method. This is better for developing and low-income countries [12].

This study aims to investigate the diagnostic accuracy of the placental thickness measured by USG in detecting IUGR babies in the third trimester of pregnancy, keeping IUGR (Parameters by using Hadlock) as the gold standard

## Materials and methods

This cross-sectional study was conducted at the radiology department of KRL Hospital from August 5, 2020, to October 25, 2021. Ethical approval was obtained from the ethical review board committee of KRL Hospital, Islamabad, vide letter reference no. KRL-HI-PUB/Aug21/04 dated August 2021.

The study included 362 pregnant women, including first-time mothers and those who had previously given birth. Informed written consent was also obtained from each patient. The ethical committee approved the study of the hospital.

Inclusion and exclusion criteria

The inclusion criteria for our study are the patients' known last menstrual period; age 20-35 years, BMI usual, and 24 weeks gestation. Patients with a history of irregular cycles, diabetes, severe renal disease, hypertension, obesity, placenta previa, and low placenta laying were excluded from this study.

Study limitations

Our study is limited to those women who underwent ultrasound examinations at 24 weeks, 32 weeks, 36 weeks, and after the birth of their child.

Data collection

The patient's complete history was taken by clinical examination and then ultrasound was carried out to measure the placental thickness (ultrasound with transabdominal 3.5 MHz probes). The fetus was checked for gross anatomical defects and viability. The ultrasound determination of gestational age was determined by calculating various growth parameters (Hadlock method) "biparietal diameter (BPD), femur length (FL), abdominal circumference (AC), head circumference (HC)."

**Figure 1:**
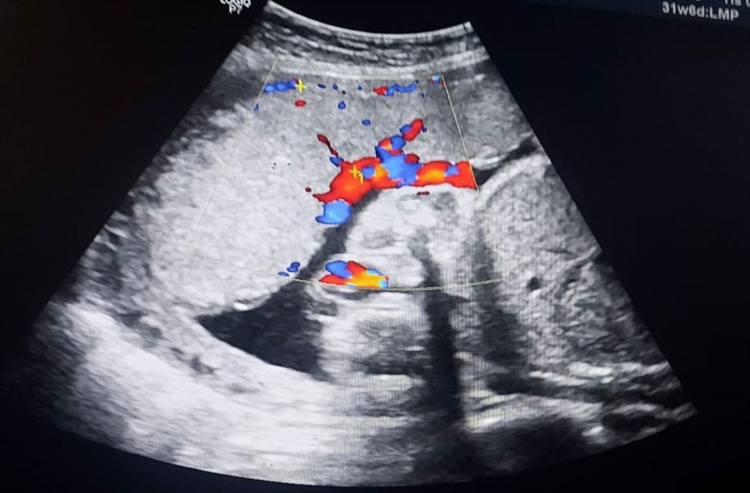
Measurement of Placental Thickness at the Level of Cord Insertion

The placental site had been determined in a longitudinal section using a two-dimensional, real-time mode. The placental thickness was measured at "the level of umbilical cord insertion in longitudinal course from lateral chorionic plate to the insertion of the cord apart from the retro placental area," as shown in Figure 1. Placental thickness was measured at 24, 32, and 36 weeks.

Data analysis

The estimated fetal weight was calculated by measuring BPD, AC, and FL using the Hadlock method on the GC logic P/7 three-dimensional machine (GE, Tampa, FL). Data were analyzed using SPSS v 23 (IBM Corp., Armonk, NY) with mean and standard deviation.

## Results

In the present study, the patient age ranged between 20 and 35 years; the mean age was 27.26 ± 4.21 years. The BMI was of the patients 26.40 ± 4.10 (kg/m^2^). The height was 170.43 ± 10.32 cm, and the weight was 75.32 ± 6.23 kg, as shown in Table 1.

**Table 1:** Basic Information of the Study Subjects BMI: Body Mass Index

Sr#	Variables	Results
	Age (years)	27.26 ± 4.21
	Height (cm)	170.43 ± 10.32
	Weight (kg)	75.32 ± 6.23
	BMI (kg/m^2^)	26.40 ± 4.10

In our study, the mean placenta thickness at 24 gestation weeks was 24.55 ± 0.79 mm, at 32 gestation weeks was 31.84 ± 1.34 mm, and at 36 gestation weeks was 35.54 ± 2.78. These results helped determine the gestational age estimation. The estimated fetal weight was at 24 weeks was 0.763 ± 0.056 kg, at 32 weeks was 1.702 ± 0.432 kg, and at 36 weeks was 2.622 ± 0.521 kg, as shown in Table 2.

**Table 2:** Placental Thickness at Different Gestation Weeks with Biometric Parameters Biparietal Diameter (BPD); Head Circumference (HC); Abdominal Circumference (AC); Femur Length (FL)

	At 24 Weeks	At 32 Weeks	At 36 Weeks
BPD (in weeks)	24.44 ± 0.81	24.44 ± 0.81	35.6.± 1.11
FL (in weeks)	24.32 ± 0.73	31.72 ± 0.86	35.43 ± 1.13
HC (in weeks)	24.11 ± 0.84	31.94 ± 0.87	35.45 ± 1.27
AC (in weeks)	23.75 ± 0.87	31.23 ± 1.14	34.61 ± 1.84
Gestational age by USG	24.43 ± 0.47	31.77 ± 0.17	35.32 ± 1.29
Placental thickness (in mm)	24.55 ± 0.79	31.84 ± 1.34	35.54 ± 2.78
Estimated fetal weight (kg)	0.763 ± 0.056	1.702 ± 0.432	1.702 ± 0.432

The results of estimated fetal weight (US) showed that 101 (86.32%) fetus of < 2.5 kg had placental thickness < 32 (mm), 16 (13.68%) fetus of < 2.5 kg had placental thickness > 32 (mm). Similarly, 33 (13.45%) fetus of > 2.5 kg had placental thickness < 32 (mm), 212 (86.55 %) fetus of > 2.5 kg had placental thickness > 32 (mm), as shown in Table 3.

**Table 3:** Cross-Tabulation of Estimated Fetal Weight Vs Measured Placental Thickness

Measured Placental Thickness (mm)	Estimated fetal Weight (US)		Total
	< 2.5 kg	>2.5 kg	
< 32 mm	101	33	134
	86.32 %	13.45 %	37.02 %
>32 mm	16	212	228
	13.68 %	86.55 %	62.98 %
Total	117	245	362
	100 %	100 %	100 %

Thus, the sensitivity of ultrasound "ability to correctly identify fetus having a low placental thickness" was 86.30%. In contrast, the specificity of ultrasound "ability to correctly identify fetus not having low placental thickness" was 86.70%. The positive predictive value "probability of having low placental thickness babies on ultrasound when it is present" was observed as 75%, and the negative predictive value "probability of having low placental thickness babies on ultrasound when it is not present" was 92%. The diagnostic accuracy of ultrasound incorrectly estimating low placental thickness was 86.40%.

## Discussion

The placenta is known as the "sprightliness" of the fetus in gestation, responsible for a wide range of functions that help the fetus mature. The placenta keeps the umbilical cord feto-maternal circulation going [8]. For average fetal growth and development, a well-developed placenta with efficient function is essential. During the fetal growth cycle, the placenta grows in size to perform its essential functions. If fetal growth is hampered, it is due to irregular placental functioning, which can be identified by the measurement of abnormal placental [13].

The thickness of the placenta is closely linked to fetal development and can play a role in the perinatal outcome. The placenta is about 3 cm thick and 15-25 cm in diameter [1].

Low birth weight neonates are predicted by a "warning limit" of 2cm placental thickness and 1.8cm placental diameter at 36 weeks. Chromosomal defects, preeclampsia, recurrent fetal infections, intrauterine growth restriction, and diabetes mellitus are linked to small placentas [14]. Diabetes mellitus placenta has been observed in circumstances such as prenatal infections, hydrops details, and diabetes mellitus. There was a significant increase in perinatal mortality and morbidity in women carrying thick placentas, which was connected to more excellent rates of fetal abnormalities, more IUGR, and more term newborns with a wide range of gestational ages [15]. Prenatal detection of IUGR is critical for a better perinatal outcome [1,11].

Our results agree with the study of Adeyekun and Ikubor [12], as they reported that study subjects were 29.1 ± 4.9 years. The mean maternal height was 1.6 ± 0.5 m and 71.4 ± 13.6 kg. The study by Nagpal et al. found the mean age of the study population was 23.1 ± 3.02 years [16]. Similarly, the study by Ahmed Shahat et al. find the mean age 25.0 ± 3.1 years, mean height 1.7 ± 0.07 m, mean weight 78.0 ± 12.0 kg, and BMI 27.1 ± 3.7 kg/m^2^ [1]. These results matched with the results of our study.

Pregnant women are subjected to similar stressors, and any maternal illness affects both the placenta and the fetus. Placental health and measures are thus able to represent the fetus's overall well-being and nutritional state and predict pregnancy outcomes. The most straightforward measurement is placental thickness, which reflects placental size [5].

Balla et al. studied the thickness of the placenta in 53 pregnant women in their third trimester [17]. They concluded that placental thickness less than 25 mm in the third trimester could indicate IUGR. In contrast, thickness greater than 45 mm could show maternal comorbidities like diabetes or hypertension or fetal anomalies such as hydrops details.

According to Damodaram et al., there is a positive association between placental volume and gestational age, but it is decreased in growth-restricted fetuses [18]. The earliest symptom of fetal growth restriction can be a decline in placental thickness for gestational age.

Because the placental thickness is nearly equal to the gestational age in weeks, it appears to be a promising indicator of fetal gestation and a predictor of fetal outcomes when measured by ultrasound. "Placental thickness below the 10th percentile has been linked to low birth weight and IUGR," says one study.

The diagnostic accuracy of ultrasound incorrectly estimating low placental thickness was 86.40%. This demonstrates that placental thickness is an outstanding predictor of IUGR. It is clear from the preceding discussion that IUGR is linked to decreased placental thickness. As a result, we believe that subnormal placental thickness can be used as an early indicator of IUGR.

## Conclusions

Between 24 and 36 weeks of pregnancy, placental thickness rises almost linearly. As a result, measuring placental thickness and other factors is critical for estimating fetal age, particularly in the late second and early third trimesters, when the exact duration of pregnancy is uncertain. Placentas that were less than 29 mm thick at 32 weeks and 31 mm thick at 36 weeks were related to higher morbidity, lower Apgar scores, and more nursery admissions. As a result, patients in this age group should give birth in a setting with appropriate nursery facilities.

Placental parameters can be measured in peripheral centers that lack Doppler and 3D ultrasound capabilities, allowing for timely referral and a stable fetus.
